# Intracellular Photodynamic Activity of Chlorin e6 Containing Nanoparticles

**DOI:** 10.16966/2470-3206

**Published:** 2016-11-17

**Authors:** Thomas Hopkins, Rahil Ukani, Raoul Kopelman

**Affiliations:** Department of Chemistry, University of Michigan. Ann Arbor, MI, USA

**Keywords:** Photo-dynamic Therapy, Nanoparticles, *Chlorin e6*, Cancer

## Abstract

Nanoparticles (NPs) containing the photo-therapeutic dye *Chlorin e6* (Ce6) have been explored in multiple studies for photo-dynamic therapy (PDT). However, little work has been carried out regarding their PDT efficacy, relative to other dye containing NPs. Here polyacrylamide nanoparticles (PAAm NPs) containing Ce6 were prepared and their PDT efficacy compared to previously reported methylene blue (MB) containing PAAmNPs. It was found that, for identical NP dosages and photon doses, the Ce6 NPs are an order of magnitude more potent in killing cancer cells.

## Introduction

Cancer is a leading cause of death in the US, with treatment mostly limited to non-selective methods, such as chemo, radio therapy, and surgery [1]. This has led to an expansive interest in researching selective methods of therapy to increase survival rates and general quality of life. The use of targeted nanoparticles has been a long-standing approach [1–3].

Hydrogel NPs have been shown to accomplish a variety of tasks in cancer treatment, such as imaging [4], visible tissue delineation [5], selective accumulation of chemo drug [6,7], photo-dynamic therapy (PDT) [8–10], photo-thermal therapy (PTT) [11], and sensing [12]. NP-mediated PDT has been of interest due to its double selectivity (cell-targeted NPs as well as laser focused irradiation) and low tumor resistance.

PDT is based on cytotoxic reactive oxygen species (ROS), produced by dyes (photosensitizers) when excited under photo-illumination in the presence of oxygen. Therefore, PDT requires light, a dye, and oxygen to have any cytotoxic effects. When contained in NPs that have been surface modified (peptides, antibodies, small molecules, etc.) to be cancer cell specific in uptake, goodselectivity in treating cancer cells can be achieved through selective accumulation [1,13].

A number of photosensitizers capable of efficient PDT have been employed, such as Photofrin, Methylene blue and *Chlorin e6. Chlorin e6* (Ce6) has been used for photodynamic therapy of both cancer and heart disease [14–18]. Here, we study the relative efficacy of Ce6 and methylene blue (MB) *in vitro,* when embedded in hydrogel, i.e., polyacrylamide NPs. Previously reported MB NPs are used for comparison [19]. Various cell lines could be used for these experiments. We chose HeLa cells because they represent a most robust cell line; it takes a sizable amount of damage/stress to kill them. This helps to illustrate good PDT efficacy by showing that even these very robust cells are being killed.

## Methods

### Materials

Ce6 is sourced from Frontier Scientific. All other chemicals were materials were sourced from Sigma Aldrich. acrylamide (AAm), amino propyl methylacrylamide (APMA), 3-(acryloyloxy)-2-hydroxypropyl-methacrylate (AHM), dioctyl sulfosuccinate sodium salt (AOT), Brij 30, 1-Ethyl-3-(3-dimethylaminopropyl) carbodiimide (EDC), N-hydroxy succinidmide (NHS), dimethyl sulfoxide (DMSO), phosphate buffer saline (PBS, 0.01 M), ammonium persulfate (APS), tetra methyl ethylene diamine (TEMED), phosphate buffer saline (PBS).

### Preparation of chlorin Ce6 hydrogel NPs

1.07 g of AOT, 2.2 mL Brij 30, and 30 mL Hexane are combined in a 100 mL round bottom flask. An aqueous phase of 28 mg APMA, 368 mg AAm, 52.6 μL AHM, 40 mg EDC, 60 mg NHS, 15mg of Ce6 100 μL DMSO, and 930 μL PBS are prepared and added to the round bottom flask. The contents of the flask are stirred for 2 hours at 500 RPM. The contents are then flushed with argon using a long neck needle in contact with the mixture for 15 minutes. Argon flow is then continued but removed from contact with the mixture. 15 mg of APS in 100 μL of water is added drop wise to the flask to initiate polymerization. 100 μL of TEMED is added drop wise and the reaction allowed to continue for 2 hours under argon. Argon is then removed and the contents of the flask exposed to oxygen to quench polymerization. Hexane is rotary evaporated and the leftover contents are cleaned in an amicon cell (300 kDa membrane) with 10×150 mL ethanol and 5×150 mL Millipore water. The final product dispersed in Millipore water is filtered using a 0.45 μm polyether sulfonate filter and lyophilized to obtain a solid product. Samples are stored in a freezer until needed.

### Singlet Oxygen Test

ROS production was tested using Singlet Oxygen Sensing Green (SOSG). A 1 mg/mL sample (2 mL, PBS) was given 10 μL of 0.5 mM SOSG in methanol and illuminated at 662 nm for 5 min. The fluorescence of SOSG was measured at 504/525 nm Ex/Em before and after illumination.

### Size analysis

Transmission electron micrographs (TEMs) were taken at the Microscopy and Image Analysis Laboratory of the University of Michigan. Samples were deposited on grids via vacuum evaporation of solvents and subsequent staining with uranyl acetate.

Blanks of NPs (no dye) were synthesized and characterized using dynamic light scattering (DLS, Delsa Nano C Particle Analyzer). DLS was not used for the active NPs, due to spectral interference.

### UV/VIS

Absorption spectra were gathered using a Shimadzu UV-1601 UV-Visible Spectrophotometer.

### Cell Culture

96-well plates were seeded with 2000 HeLa cells per well (n = 16) containing 200 μL of cell culture media. Plates of light and dark toxicity were given NP dosages of 0 and 200 μg/mL; 0 μg/mL were control groups that defined 100% viability. Cell viability was determined colorimetrically, in a plate reader, via MTT assay [13]. Briefly, cell media were replaced with colorless media, containing no serum (100 μL), and 20 μL of 5 mg/mL MTT reagent and incubated for 4 hours. The media were then carefully removed and the formazan crystals solubilized using 100 μL of DMSO.

### Light Toxicity

A 96-well plate was illuminated using an LED array (625 nm ± 20 nm, 35.2mW) for 6 min.

## Results

**Figure 1:**
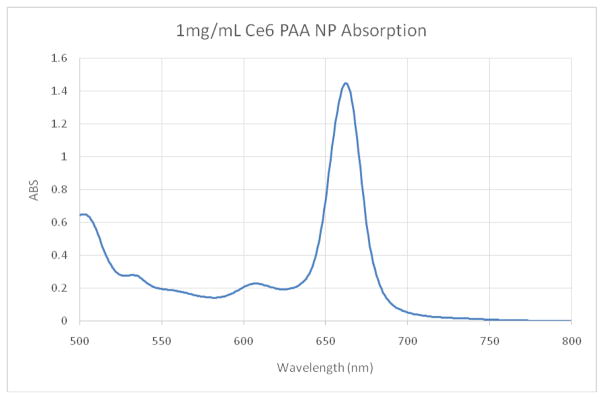
Absorption spectra of the as prepared Ce6 NPs. Concentration = 1 mg/mL in PBS.

**Figure 2:**
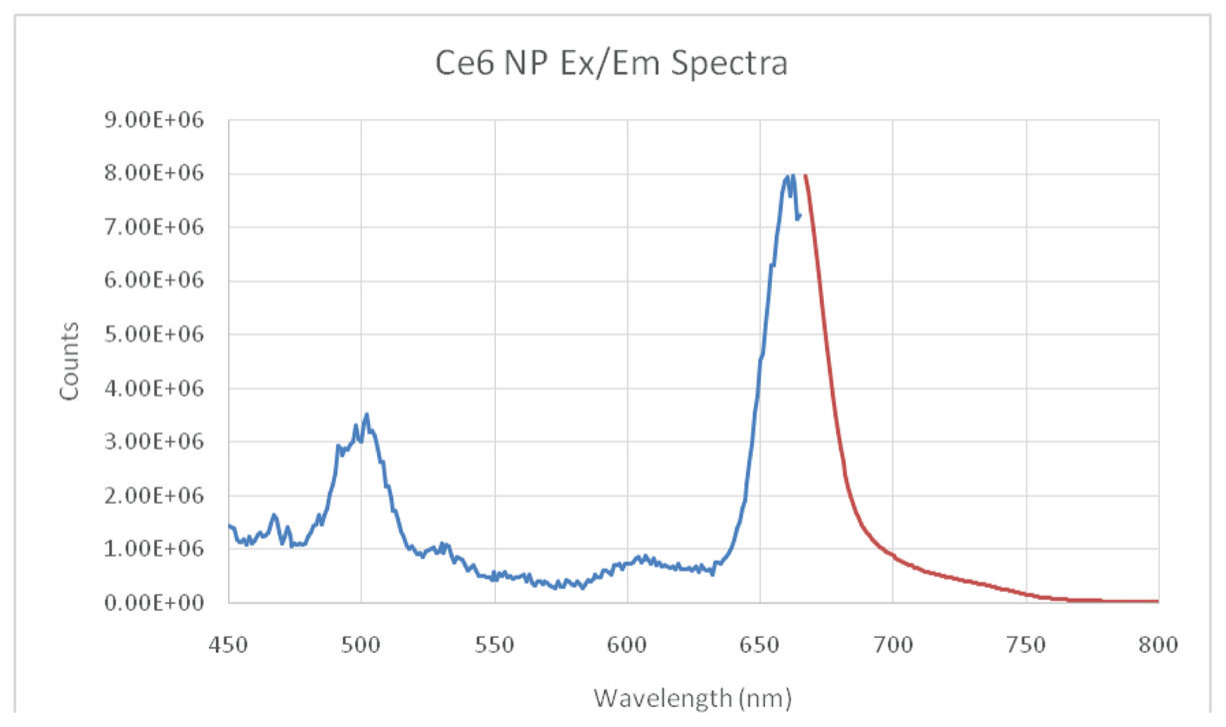
Ce6 hydrogel NP Excitation (blue)/Emission (red) at 0.1 mg/mL concentration in PBS. Excitation spectra taken using 668 nm fluorescence.

**Figure 3:**
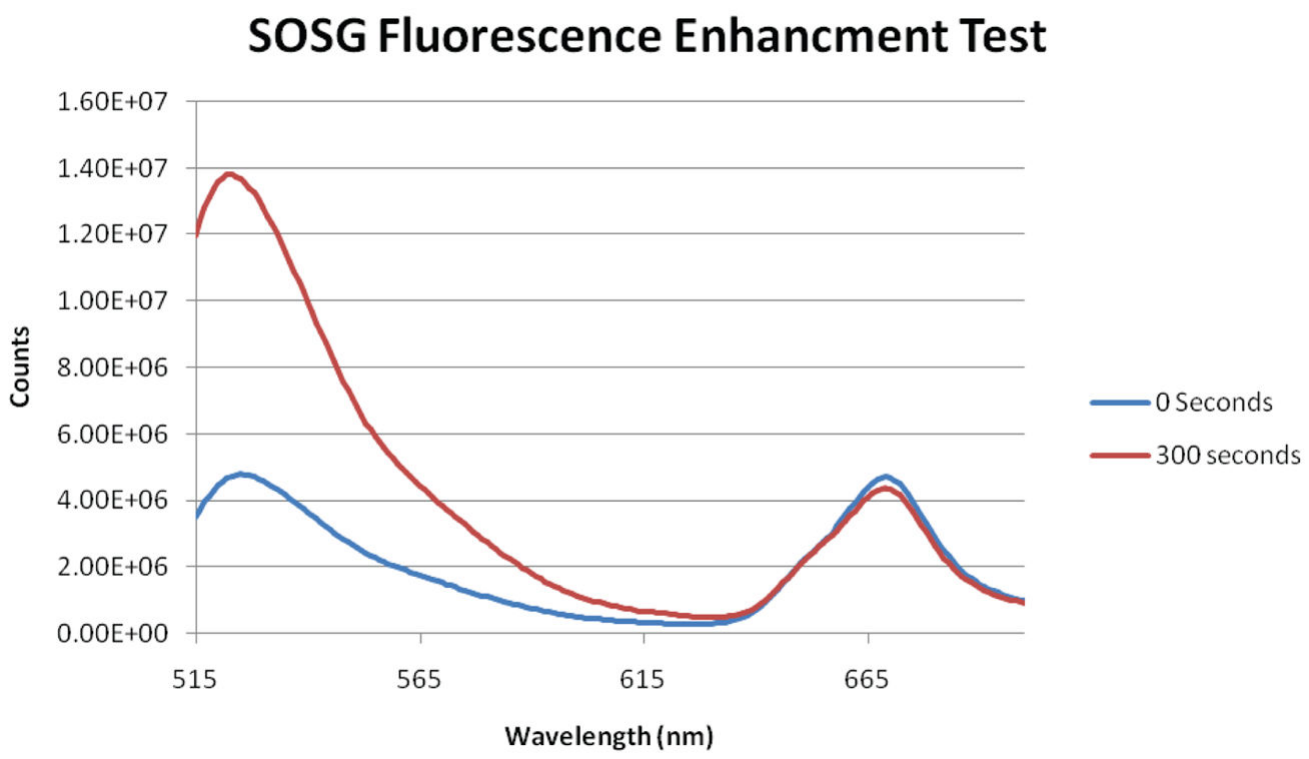
SOSG Fluorescence Enhancement Assay. The peak at ~668 nm is due to Ce6 fluorescence.

**Figure 4:**
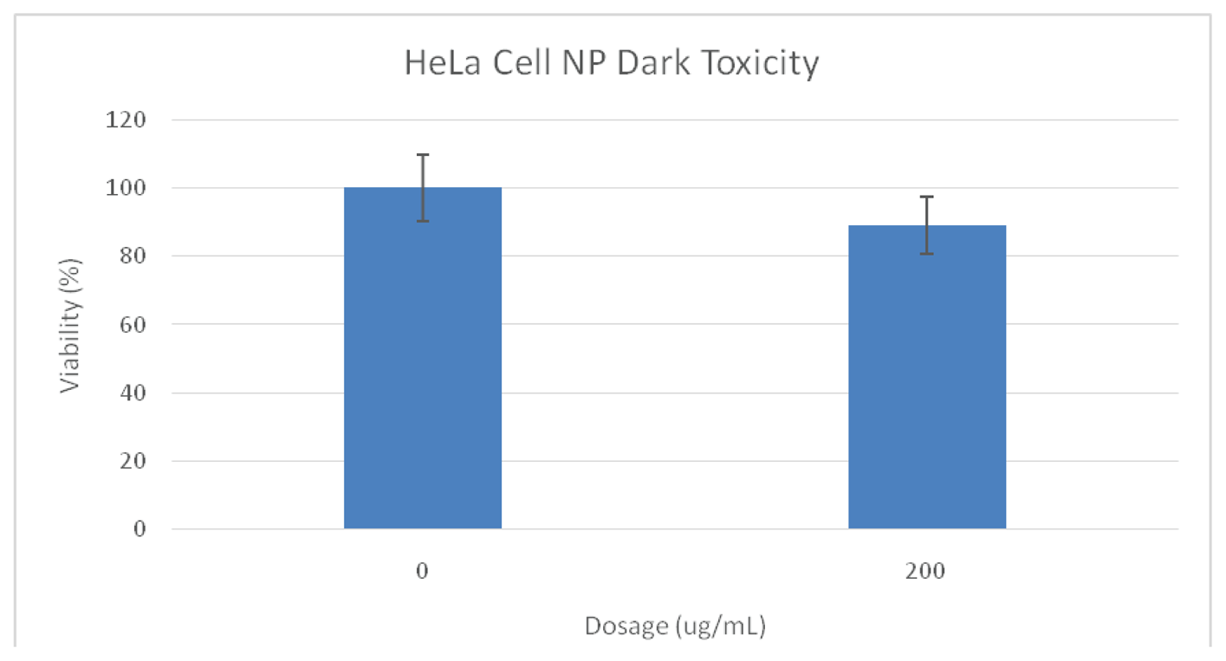
MTT assay results of Ce6 NP dark toxicity plate. Cells were 89% viable at a dosage of 200 ug/mL.

**Figure 5:**
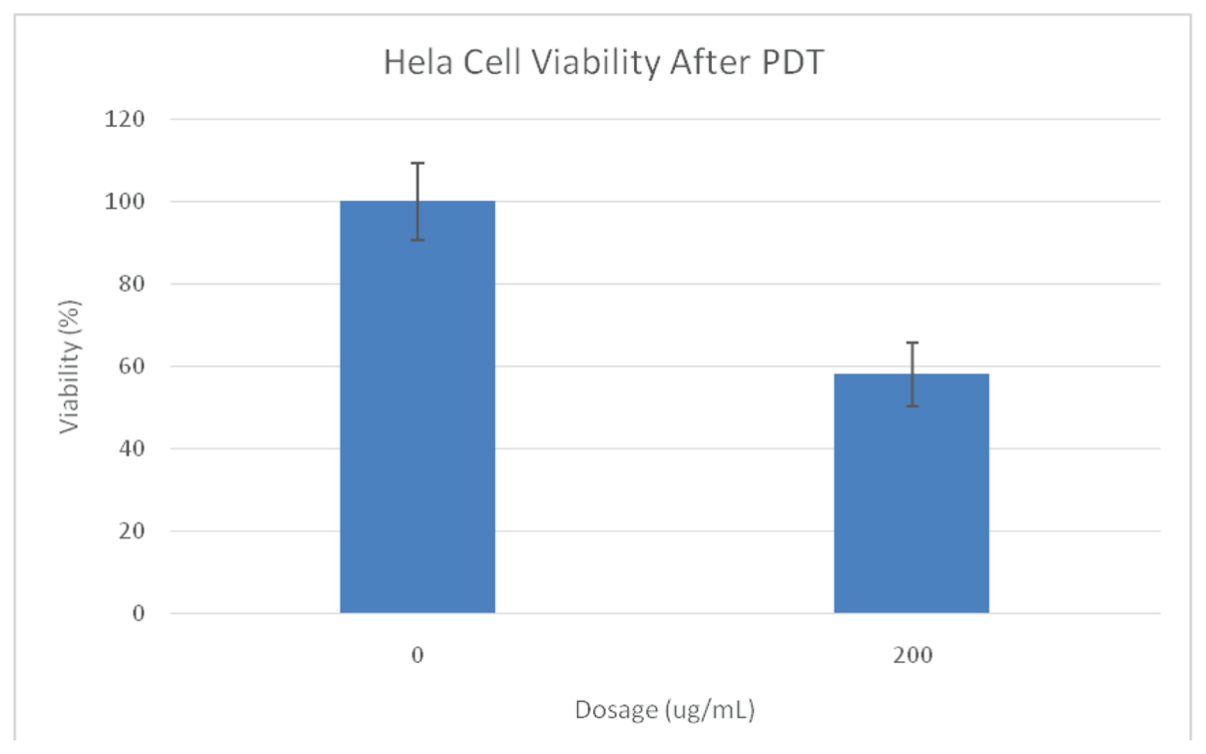
MTT assay results of Ce6 NP light toxicity plate containing HeLa cells. The cells were 58% viable after 6 min of photo-illumination at a dosage of 200 ug/mL Ce6 NPs. Illumination time=6 minutes, 35.2 mW LED array (625 nm ± 20 nm).

**Figure 6:**
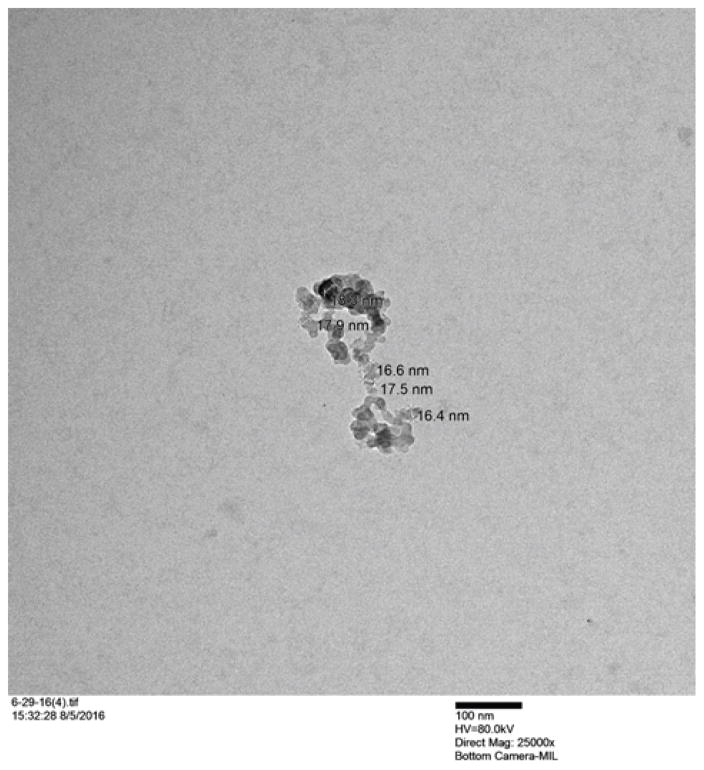
TEM image of dehydrated Ce6 hydrogel NPs.

**Figure 7:**
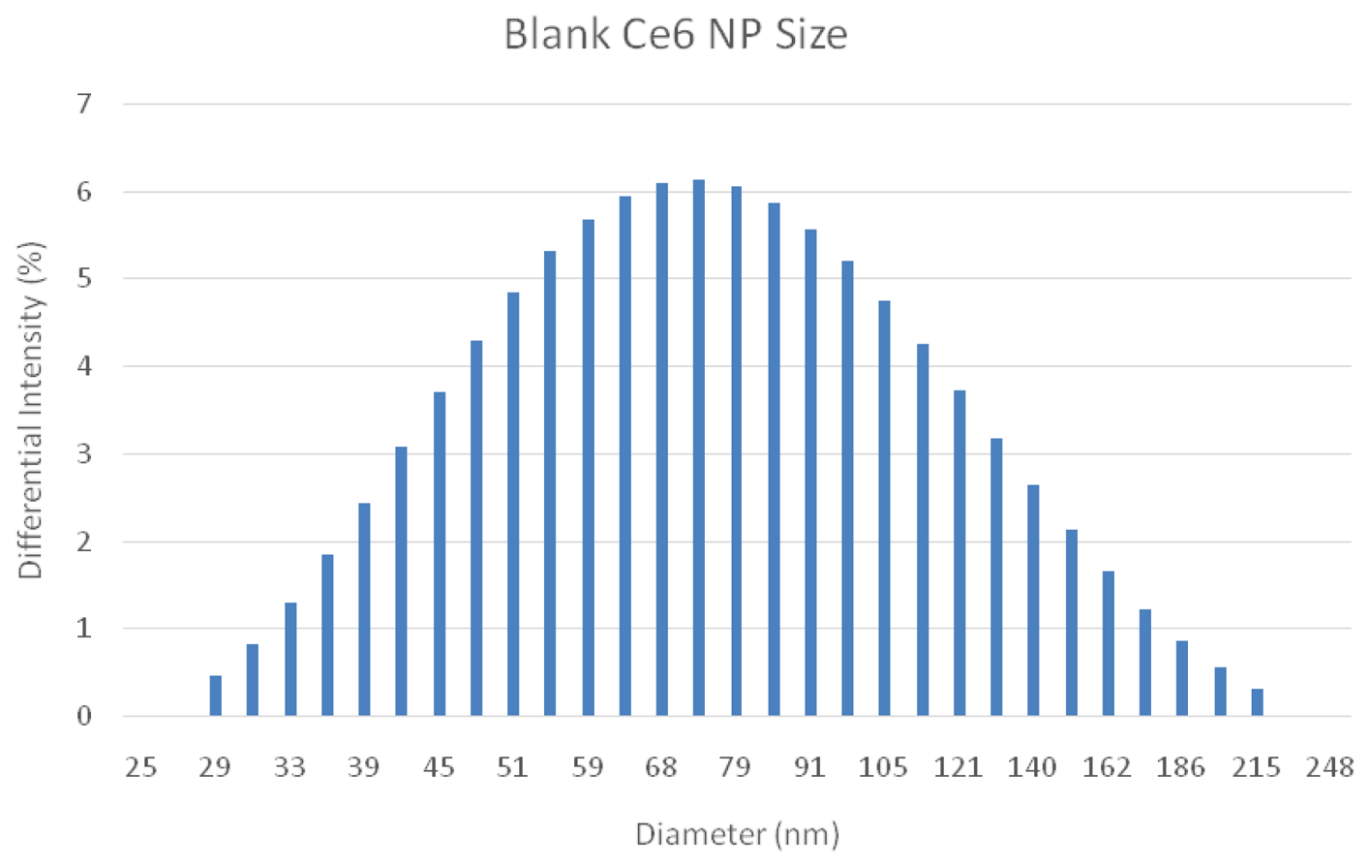
DLS analysis of hydrated Ce6 NP Blanks. Average Diameter =68 nm, PDI=0.204.

Results were shown in Table 1 and figures(1–7).

## Discussion

Ce6 is moderately hydrophobic and so tends to aggregate in saline solutions. The UV/VIS absorption spectrum shows a dominant peak at ~662 nm, characteristic of Ce6 in the monomeric form (Figure 1) [15]. Strong fluorescence at 668 nm was also detected, a typical position of monomer Ce6 (Figure 2) [15]. This indicates that the NP suitably protects Ce6 from aggregation. The loading is estimated to be ~23–24 nmol Ce6 per mg of NP using an extinction coefficient of 61,000 and 662 nm peak.

SOSG was employed as a boolean test to determine if the ROS production capability of Ce6 had been maintained after conjugation and encapsulation. SOSG is highly selective to detection of singlet oxygen, appearing as an enhancement of the fluorescence signal. (Figure 3) shows a strong and definitive fluorescence enhancement after illumination with Ce6 NPs, confirming the preservation of the capacity of Ce6 to produce ROS.

The dark toxicity 96-well plates of cells with Ce6 NPs demonstrated good biocompatibility; approximately 89% of the cells were viable after incubation for 24 hours in the dark (Figure 4). TEMs showed the dehydrated NPs to be ~17 nm (Figure 6), similar in size to the ~14 nm MB NPs studied [19]. Blanks of the NPs (no dye) were synthesized as a secondary size characterization method. The diameter of these blank NPs was found to be about 68 nm, a size typical to biocompatible nanoparticles (Figure 6) [19].

The light toxicity plate showed only 58% viability after illumination for 6min with the LED source (Figure 5). We point out that it is known, for the NPs used, that they will enter cells through endocytosis, with saturation occurring within 24 hours [20]. Coupled with the SOSG test (Figure 2), this large difference in viability between light and dark plates is attributed to cell kill by PDT.

Previous work with the MB NPs using the same illumination source and dosages resulted in 70% viability (Table 1) [19]. However, the MB NPs are significantly more optically absorbent in the spectral range of illumination, at about 625 nm (wavelength of peak illumination). Specifically, these MB NPs have an OD=0.5, vs. an OD=0.2 for Ce6. Thus the Ce6 NPs displayed a higher cell kill rate than the MB NPs, while being significantly less absorbing (Table 1). This indicates that for this particular system, if the Ce6 NPs were employed using their peak wavelength of absorption (662 nm), they would be about an order of magnitude more potent than the reported MB NPs.

The only observed disadvantage of the Ce6 hydrogel NPs, compared to the MB hydrogel NPs, is the overall decreased NP hydrophilicity with increased Ce6 loading. Future work with Ce6 should focus on increasing the hydrophilicity of its nano platform, so that proper concentrations of NPs can be achieved for applications in animal and, eventually, human models.

## Conclusion

The results presented here show that the as prepared Ce6 hydrogel NPs have good biocompatibility and display greater efficacy in killing cancer cells, compared to the previously used MB NPs. This information should be useful for selecting the most PDT effective photosensitizer containing nanoparticles, as will be demonstrated in forthcoming work on animal models.

## Figures and Tables

**Table 1:** Comparative Data of Ce6 and MB NPs [19]. OD = Optical Density at peak wavelength of light source, NP concentration = 1 mg/mL. Note: Same light source and configuration used for both photosensitizers.

NPs	Wt% Dye Loading	nmol dye/mg NP Loading	TEM (nm)	NP OD @ ~625 nm	6 min PDT Viability
Ce6	1.40	23.4	17	0.2	58%
MB	0.63	13.1	14	0.5	70%
